# Brain microvascular endothelial cells differentiated from a Friedreich’s Ataxia patient iPSC are deficient in tight junction protein expression and paracellularly permeable

**DOI:** 10.3389/fnmol.2025.1511388

**Published:** 2025-04-15

**Authors:** Frances M. Smith, Daniel J. Kosman

**Affiliations:** Department of Biochemistry, Jacobs School of Medicine and Biomedical Sciences, The State University of New York at Buffalo, Buffalo, NY, United States

**Keywords:** Friedreich’s Ataxia, blood–brain barrier, tight junction, Claudin-5, Occludin, ZO-1, transendothelial electrical resistance, mitochondrial energy metabolism

## Abstract

Friedreich’s Ataxia (FA) is a rare, inherited ataxia resulting from GAA triplet expansions in the first intron of the Frataxin (FXN) gene, which encodes a mitochondrial protein involved in the incorporation of iron into iron–sulfur clusters. We previously identified decreased levels of F-actin and tight junction (TJ) proteins, which coincided with paracellular permeability in an FXN shRNA-mediated knockdown immortalized human brain microvascular endothelial cell (BMVEC) model. This premise is underexplored in the FA literature, prompting us to confirm these findings using a patient-derived iPSC model. One line each of FA patient iPSCs and age- and sex-matched *apparently healthy* iPSCs were differentiated into BMVEC-like cells. We quantified actin glutathionylation, F-actin abundance, TJ expression and organization, and barrier integrity. In the absence of dysregulated F-actin organization, FA iBMVEC exhibited a loss of 50% ZO-1, 63% Occludin, and 19% Claudin-5 protein expression, along with a disruption in the bi-cellular organization of the latter two proteins. Functionally, this correlated with barrier hyperpermeability, delayed barrier maturation, and increased flux of the fluorescent tracer Lucifer Yellow. These data indicate that decreased barrier integrity is a pathophysiological phenotype of FA brain microvascular endothelial cells. Clinically, this may represent a targetable pathway to reduce brain iron accumulation, neuroinflammation, and neurodegeneration profiles in FA. Additionally, an investigation into other barrier systems, such as the blood–nerve barrier, blood-CSF barrier, or cardiac vasculature, may provide insights into the extra-neural symptoms experienced by FA patients.

## Introduction

Friedreich’s ataxia (FA) affects approximately 1 in 50,000 US citizens, disproportionately affecting the Caucasian population (Indelicato et al., 2020). FA is the most commonly inherited ataxia in Europe, the Middle East, South Asia (the Indian subcontinent), and Northern Africa (Bidichandani et al., 2024). Additionally, the incidence of FA is lower than average in Mexico and remains undocumented in Southeast Asia, Sub-Saharan Africa, and among Native Americans (Bidichandani et al., 2024).

FA patients are diagnosed on average at the age of 15 and experience continual disease progression until a mean age of death of 37 (Payne and Wagner, 2012; Patel et al., 2016). FA is a progressive neuromuscular disease characterized by many neurological phenotypes, including, but not limited to, ataxia, dysphagia, weakness, spasticity, loss of hearing and vision, and peripheral neuropathy (Koeppen et al., 2016). Indeed, the dorsal root ganglion is recognized as the major site of disease, as evidenced by the selective vulnerability of large neurons (Bidichandani et al., 2024; Koeppen et al., 2009). Much of the FA literature focuses on the pathology of cardiomyocytes, with approximately 60% of patients succumbing to the disease due to fibrosis of the left ventricular wall and eventual hypertrophic cardiomyopathy (Jensen and Bundgaard, 2012). However, many tissue systems and alternative pathologies of the disease remain to be investigated.

A GAA expansion repeat tract in the first intron of the Frataxin (FXN) gene decreases transcriptional elongation and protein expression, representing the disease mechanism (Campuzano et al., 1997; Campuzano et al., 1996). FXN is a mitochondrial protein required for assembling iron–sulfur clusters, and its low expression in FA causes mitochondrial iron accumulation. This affects energy metabolism as the first four complexes of the electron transport chain contain iron prosthetic groups, leading to deficits in oxidative energy metabolism (Smith and Kosman, 2020).

To compensate for perceived iron starvation, the cell responds via the iron starvation hypothesis, increasing expression of cell surface iron importers and decreasing expression of the iron export protein, Ferroportin. This leads to further intracellular iron sequestration (Grander et al., 2024; Huang et al., 2009) and results in the production of reactive oxygen species and oxidative stress, compounded by decreased expression of the major antioxidant transcription factor Nrf2, which regulates over 2,000 antioxidant activities (Paupe et al., 2009; McCord et al., 2023).

Oxidative stress was an early identified pathophysiology in FA patient fibroblasts, conferring the post-translational modification actin glutathionylation (Pastore et al., 2003; Piemonte et al., 2001). This is a reversible oxidative modification at Cys374, the penultimate cysteine in the actin-actin binding domain of actin monomers (Dalle-Donne et al., 2003). Thus, this modification stunts the rate, extent, and stability of filamentous actin (F-actin) formation. However, the investigation of how these cytoskeletal changes may affect diverse tissue systems in disease has never been examined, despite the importance of cytoskeletal architecture in barrier-forming cells.

The actin cytoskeleton anchors tight junction (TJ) proteins, which are transmembrane proteins at the apical surface of vascular barrier cells. Importantly, TJs are the only permeability-governing junctions that rely on actin anchorage for their function. In an FXN-deficient immortalized brain microvascular endothelial cell (BMVEC) line, we previously identified a decrease in F-actin and TJ protein expression coinciding with increased barrier permeability (Smith and Kosman, 2024). Thus, we sought to validate these findings in a more clinically relevant cell type, FA patient-derived iPSCs differentiated into brain microvascular endothelial-like cells (iBMVEC). These cells comprise the blood–brain barrier (BBB), the network of vasculature that governs the selective permeability of circulatory solutes into the brain interstitium. This tissue system is essential for maintaining proper sterility, nutrient balance, health, and central nervous system homeostasis.

Such a hypothesis is intriguing, especially considering the progressive increases in cerebellar brain iron accumulation observed in the FA patient, distinct from atrophy (Ward et al., 2019). This accumulation over time suggests either increased iron influx across the BBB or decreased efflux from the cerebrospinal fluid barrier. However, there is a discrepancy regarding the iron content in the brain, as post-mortem staining of ferrous iron reveals no difference in total cerebellar brain iron content between FA patients and controls (Koeppen et al., 2007). Since this is a postmortem study that lacks detection of ferrous iron, further investigation is necessary to fully understand iron homeostasis in the brains of FA patients.

Indeed, increased BBB permeability is a consequence of several other neurodegenerative diseases, including Parkinson’s, Alzheimer’s, and Huntington’s diseases, increasing the vulnerability of the neuroenvironment to systemic acute-phase signals (Gray and Woulfe, 2015; Alkhalifa et al., 2023; Di Pardo et al., 2017). Thus, targeting this pathological feature in FA could be beneficial in alleviating brain iron accumulation, neuroinflammation, and neurodegeneration experienced by patients throughout the course of disease progression.

To investigate this premise, we used two iPSC lines differentiated into brain microvascular endothelial-like cells (iBMVEC): one line from an FA patient and one line from an age-, race-, and sex-matched assumed healthy control (HC). While our sample size is a limitation to the statistical robustness achievable with a larger group, we chose to examine alterations to specific pathways to inform future therapeutic investigations.

We quantified significant reductions in the expression of the TJ proteins Claudin-5, Occludin, and ZO-1 and found decreased concentrations of Claudin-5 and Occludin at the bi-cellular interfaces of adjacent cells. These decreases were accompanied by significantly lower barrier resistance to ion flux and increased permeability to the paracellular tracer Lucifer Yellow. Together, these data indicate that FXN-deficient cells have inherent decreases in TJ protein expression, which is detrimental to barrier function. This provides molecular insight into the microvascular homeostasis of FA patients and encourages the investigation of other barrier systems in the disease. Additionally, the experiments reported here could be used to screen compounds relevant to improving barrier physiology, both in the context of FA and other diseases of the neurovascular unit.

## Materials and methods

### Reagents

All chemical reagents were obtained from Sigma Aldrich unless otherwise indicated. Lyophilized compounds are dissolved according to manufacturer instructions. All protocols are conducted per manufacturer instructions unless otherwise noted.

### Antibodies

Primary: Rabbit α-FXN (ThermoFisher #PA5-13411, used at 1:500), Mouse α-TATA Binding Protein (ThermoFisher #49–1,036, used at 3.2 μg/ml), Rabbit α-Nrf2 (AbClonal #A21176, used at 1:750), Rabbit α-claudin-5 (Abcam #ab131259, used at 1:1,000), Rabbit α-occludin (Abclonal #A2601, used at 1:1,000), Rabbit α-ZO-1 (Abclonal #A0659, used at 1:5,000), Rabbit α-β-actin (Cell Signaling Technologies #8457, used at 1:5,000), Rabbit GLUT-1 (Abclonal #A6982, used at 1:1,000), and Rabbit PECAM-1 (Abclonal #A0378, used at 1:1,500). All primary antibodies were diluted in 1% milk-TBST.

Secondary: Donkey α-goat: 647 (Thermo Fisher #A21447, used at 1:1,000), Donkey α-rabbit: 488 (Thermo Fisher #A21206, used at 1:1,000), Goat α-mouse: HRP (Novus Biologicals NBP2-31347H, used at 1:5,000), and Goat α-rabbit: HRP (Cell Signaling Technologies #7074, used at 1:5,000). All secondary antibodies were diluted in 3% milk-TBST. All chemiluminescent antibodies were activated using ECL reagents in 1–5 min (Clarity Max, Biorad). All blot imaging was performed on the ChemiDoc Illuminator (Biorad).

### Cell culture—iPSC

iPSCs derived from a 34-year-old male patient with FA (line #USFi001-A) were generously provided by Dr. Thomas McDonald at the University of South Florida. This patient was previously characterized as having 1,167 GAA repeats in FXN allele 1 and 834 repeats in allele 2 (Angulo et al., 2021). iPSCs from a healthy 49-year-old white male (line #AG28851) were obtained from the Coriell Institute for Medical Research. Cultures were maintained on Matrigel-coated (Corning) tissue culture plates in mTESR+ (Stem Cell Technologies) and supplemented with 10 μM Y27632 (iXCells Biotechnologies) for 24 h after passaging.

### Differentiation to iBMVEC

**Figure 1:**
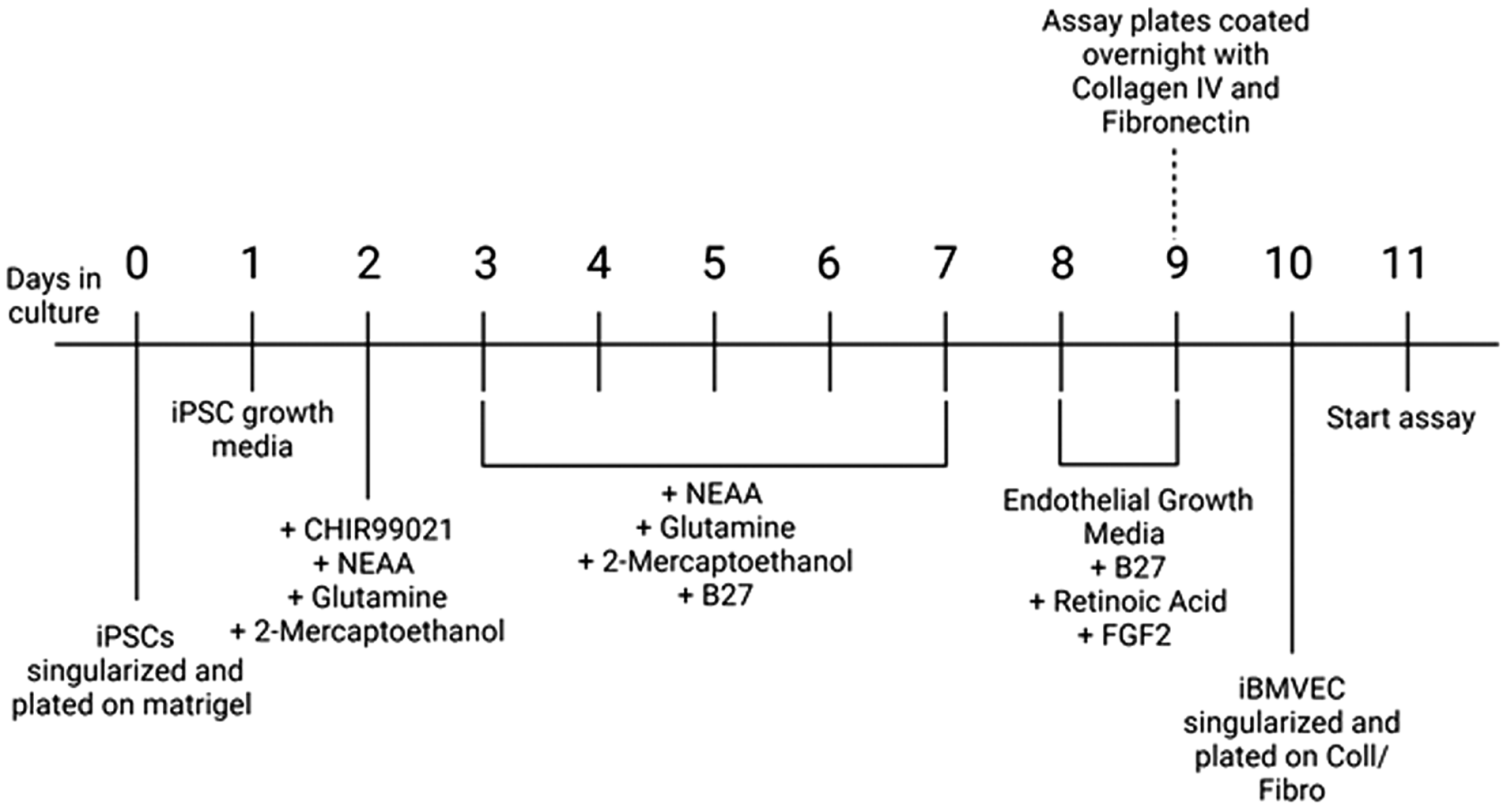
Differentiation protocol. A schematic of differentiation from iPSC to iBMVEC as described in (Foreman et al., 2023).

Differentiation into iBMVEC-like cells followed an established protocol, as represented schematically in Figure 1 (Foreman et al., 2023). Briefly, iPSCs, passaged at least three times in culture, were resuspended in mTESR+ medium supplemented with 10 μM Y27632 to form a single-cell suspension and plated at 200–300,000 cells per well on Matrigel-coated 6-well tissue culture plates. On day 1, post-plating, the media was changed to mTESR+. On day 2, the medium was replaced with DeSR1 (DMEM/F12 ThermoFisher), 1X MEM non-essential amino acids (Corning), 0.5X GlutaMAX (Corning), and 0.1 mM Beta-mercaptoethanol) supplemented with 6 μM CHIR99021. For the following 5 days, the medium was changed daily to DeSR1 supplemented with 1X B27 (ThermoFisher). On day 9 post-plating, the medium was switched to HECSR+ (Human Endothelial Serum-Free Media (HESFM, ThermoFisher) supplemented with 1X B27, 10 μM retinoic acid (ThermoFisher), and 20 ng/ml FGF2 (Abcam). On day 10 post-plating, tissue culture plates were coated with 20 μg/ml fibronectin (Corning) and 80 μg/ml Collagen IV (Advanced Biomatrix) or with 400 μg/ml collagen IV and 100 μg/ml fibronectin for transwell inserts in water for 24 h at 37°C. On day 11 post-plating, cells were dissociated using Accutase (ThermoFisher) and replated in HECSR+ containing retinoic acid and FGF2. Experiments were conducted 1–6 days after the second plating.

### Western blotting

iBMVEC were lysed in RIPA buffer containing 4X protease inhibitor (Promega), and the lysates were spun at 13,000 × *g* for 15 min at 4°C. Supernatants were used to quantify protein content using the BCA assay according to the manufacturer’s instructions (ThermoFisher). For FXN, TATA-binding protein (TBP), and Claudin-5, 15–35 μg of lysate were electrophoresed through a 12% Bis-Tris Bolt gel (ThermoFisher), transferred to water-activated nitrocellulose membranes, and blocked for 10 min at RT in EveryBlot Blocking Buffer (Bio-Rad).

### Western blotting with stain free gels

For Nrf2, ZO-1, and Occludin, 15–35 μg of lysate were electrophoresed through a 4–20% Stain Free gel (Bio-Rad). The gel was then activated with UV illumination for 1 min using the Bio-Rad GelDoc. Proteins were transferred to ethanol-activated PVDF, and total protein was quantified through UV illumination on the GelDoc. The membrane was blocked as previously described.

All membrane transfers were conducted using the “Mixed Molecular Weight” pre-defined setting of the Bio-Rad TurboBlotter. Antibody catalog numbers and dilutions are detailed in Supplementary Table S1. All primary antibodies were incubated overnight in 1% milk/TBST at 4°C, followed by three washes with TBST for 10 min. All secondary antibodies were incubated in 3% milk/TBST for 1 h at RT, followed by three washes with TBST for 10 min. All blots were imaged using the Bio-Rad ChemiDoc. Secondary antibodies conjugated to HRP were incubated for 1 to 5 min with ECL Clarity Max (Bio-Rad) for chemiluminescent detection.

### Actin glutathionylation pulldown

iBMVEC were lysed in lysis buffer (50 mM Tris–HCl pH 8, 137 mM NaCl, 2 mM EDTA, and 1% Triton-X) with 4X protease inhibitor (Promega), spun, and quantified for protein concentration as described. Lysate (15 μg) was taken for the “input” gel. Lysate (150 μg) was transferred to a clean tube and pre-cleared using A/G agarose beads at a final dilution of 1:20. Samples were rotated for 30 min at 4°C, followed by one spin at 1,000xg for 3 min. The pre-cleared supernatant was used for pulldown. Separately, 20 μl of A/G agarose beads per reaction were added to 20 μl mouse anti-glutathione primary antibody. The reaction was rotated for 1 h at 4°C. A “beads only” control for one sample each of HC and FA iBMVEC was rotated in the absence of the primary antibody to assess non-specific binding. At the end of the incubation, beads were washed twice in lysis buffer and spun at 2,000xg for 2 min. Beads (40 μl) were transferred to tubes containing 150 μg of pre-cleared lysate. Reactions were rotated overnight at 4°C. Reactions were spun and washed three times in wash buffer (10 mM Tris–HCl pH 7.4, 1 mM EDTA, 137 mM NaCl, 1% Triton-X) at 2,000xg for 2 min. 2X Laemmli buffer with 100 mM DTT was added per sample and heated at 50°C for 10 min. The reactions were spun once more, and the lysate was electrophoresed on a stain-free gel and transferred to PVDF as described. A gel including the input protein was run alongside and blotted under the same conditions, both probing for rabbit-anti-β-actin. Densitometry of the immunoprecipitated bands was normalized to that of the input blot and further normalized to total protein.

### Agilent seahorse Mito stress test

iBMVEC were plated at 35,000 cells per well on a Collagen/Fibronectin-coated Seahorse XF96 Plate. The assay began 24 h later, following all manufacturer instructions. After completion, the wells were lysed in NP40 lysis buffer and quantified for total protein content. All values used are normalized to mg of protein per well.

### Plate-based assays: Ferro Orange, MitoFerroGreen, and MitoTracker green

iBMVEC were seeded at 190,000 cells per well in 24-well plates coated with collagen and fibronectin. After 24 h post-plating, the cells were incubated with 1:1,000 Ferro Orange (Dojindo), 1:200 MitoFerro Green (MFG, Dojindo), and 0.7 μg/ml Hoechst, or with 1:10,000 MitoTracker Green (MTG, ThermoFisher) and 0.7 μg/ml Hoechst in HBSS for 30 min. Ferro Orange was quantified using 544 nm excitation and 590 nm emission prior to washing. All other stains were quantified following three HBSS washes according to manufacturer instructions: 480 nm excitation and 520 nm emission for MFG and MTG, and 355 nm excitation and 460 nm emission for Hoechst. All fluorometric values were normalized to Hoechst.

### Indirect immunofluorescence: Nrf2, TfR, Fpn, ZO-1, Occludin, and Claudin-5

iBMVEC cells were plated onto glass coverslips coated with collagen and fibronectin. After 24 h post-plating, the cells were fixed in 3.7% paraformaldehyde in PBS, rocking for 10 min at RT. The cells were then blocked and permeabilized in 0.1% BSA, 0.1% Tween-20, and 0.3 M glycine in PBS, rocking for 1 h at RT. All primary and secondary antibodies were diluted to 1:1,000 in 1% BSA; the catalog numbers are listed in Supplementary Table 2. Coverslips were incubated with all primary antibodies overnight at 4°C, washed three times in PBS for 5 min each, and then incubated with secondary antibodies for 1 h at RT. Hoechst-33342 (0.7 μg/ml) was diluted in PBS and incubated with the cells for 20 min at RT. The coverslips were washed three more times and then mounted on glass slides using Prolong Gold Antifade (ThermoFisher).

Images were captured using 40X magnification for Nrf2, TfR, and Fpn, and 63X oil immersion magnification for ZO-1, Claudin-5, and Occludin, all on the Leica DMi8 inverted microscope. Total staining of TfR and Fpn was normalized to Hoechst. Staining of Nrf2 was quantified by drawing a region of interest around 2 to 5 cells per image for each condition, normalizing the staining intensity to the area of the region of interest. Nuclear Nrf2 was assessed by outlining the nucleus and normalizing the staining intensity to the area of that outline. The percentage of Nrf2 in the nucleus was calculated by dividing nuclear staining intensity by whole cell intensity and converting it to a percentage. Staining quantification at adjacent membranes for ZO-1, Claudin-5, and Occludin was conducted by outlining regions of interest around bicellular interfaces and normalizing staining intensity for each selected area.

### Phalloidin-Texas red staining

Texas Red Phalloidin was used to evaluate F-actin abundance. Cells were grown, fixed, permeabilized, and blocked, as previously mentioned. Phalloidin was diluted to 1:400 in PBS with 0.7 μg/ml Hoechst and applied to the cells, rocking at RT for 20 min. The cells were washed and mounted according to prior protocols. The intensity of total cell and bicellular membrane staining was quantified as described for the TJ proteins. Membrane and cortical actin ring (CAR) staining were analyzed as detailed in our previous work (Smith and Kosman, 2024). Briefly, a 7.44-μm line was drawn through the membrane of a single cell (not at bicellular interfaces), positioning the extracellular space on the left, the start of the plasma membrane in the middle of the drawn line, and the intracellular space on the right. Pixel intensity was quantified using a histogram along the drawn line (Dagda et al., 2009). At least three membrane regions of interest were quantified per image, and all lines averaged per condition. 300 nm intracellular from the start of the plasma membrane (+100 nm for to account for error) is shaded to represent the cortical actin ring (CAR).

### Barrier permeability

iBMVECs were seeded at 170,000 cells per well in a 0.1 μm pore size, 24-well transwell format (Greiner) using HECSR+ with retinoic acid and FGF2. A transwell without cells was included in all experiments [cell-free blank, (CFB)], and its resistance value was subtracted from each sample well. Transendothelial electrical resistance (TEER) was measured at 24-h intervals from 24 to 96 h post-plating using the EndOhm-6G (World Precision Instruments). Lucifer Yellow (LY, 50 μM) was added to the apical chamber and incubated at 37°C for 45 min. Basal media aliquots were removed and analyzed for LY flux at 428 nm excitation and 536 nm emission, with absolute flux determined by reference to a standard curve.

### Hoechst Transwell staining

iBMVECs were seeded on transwells as described. One cell-free blank was assessed after 24 h. TEER was measured at 24-h intervals as previously described. The media in each chamber was changed at 24-h intervals to replicate the permeability experiments. At the appropriate time point, transwells were washed three times in PBS, fixed in 3.7% paraformaldehyde for 10 min at RT, and washed three more times in PBS. Transwells were incubated apically in 0.7 μg/ml Hoechst for 10 min at RT and washed three additional times in PBS. A drop of Prolong Gold Antifade mounting media (ThermoFisher) was added to a glass microscope slide (Globe Scientific). The transwell mesh was carefully removed from the outer cup and placed on top of the mounting media with the apical membrane of the cells facing down. Another drop of mounting media was added to the transwell mesh, and a glass coverslip was placed on top. Slides were allowed to dry at RT overnight and were kept at 4°C, protected from light, until used for imaging.

### Statistics

Prism 5 (GraphPad) was used for statistical analyses and graphical representation. Data points considered outliers were those that fell outside two standard deviations from the mean and were excluded from the analysis. A 95% confidence interval was used for all analyses. The F-test determined whether the sample groups had significantly different standard deviations. If they did, Welch’s correction was applied with a confidence interval of 95%. Timed experiments, including TEER and Lucifer Yellow flux, were analyzed using two-way ANOVA at a 95% confidence interval. *Biological replicates* indicate the number of independent iBMVEC differentiations.

## Results

### FXN expression is decreased in FA iBMVEC

iPSCs derived from a 49-year-old apparently healthy male and a 34-year-old male with FA have differentiated into brain microvascular endothelial cell-like cells (iBMVEC), as previously described (Foreman et al., 2023). A schematic of the differentiation protocol is shown in Figure 1. The successful differentiation of iPSCs into iBMVECs was validated through Western blotting of the EC markers PECAM-1 and GLUT-1 (Supplementary Figures 1A,B) (Lippmann et al., 2012).

**Figure 2:**
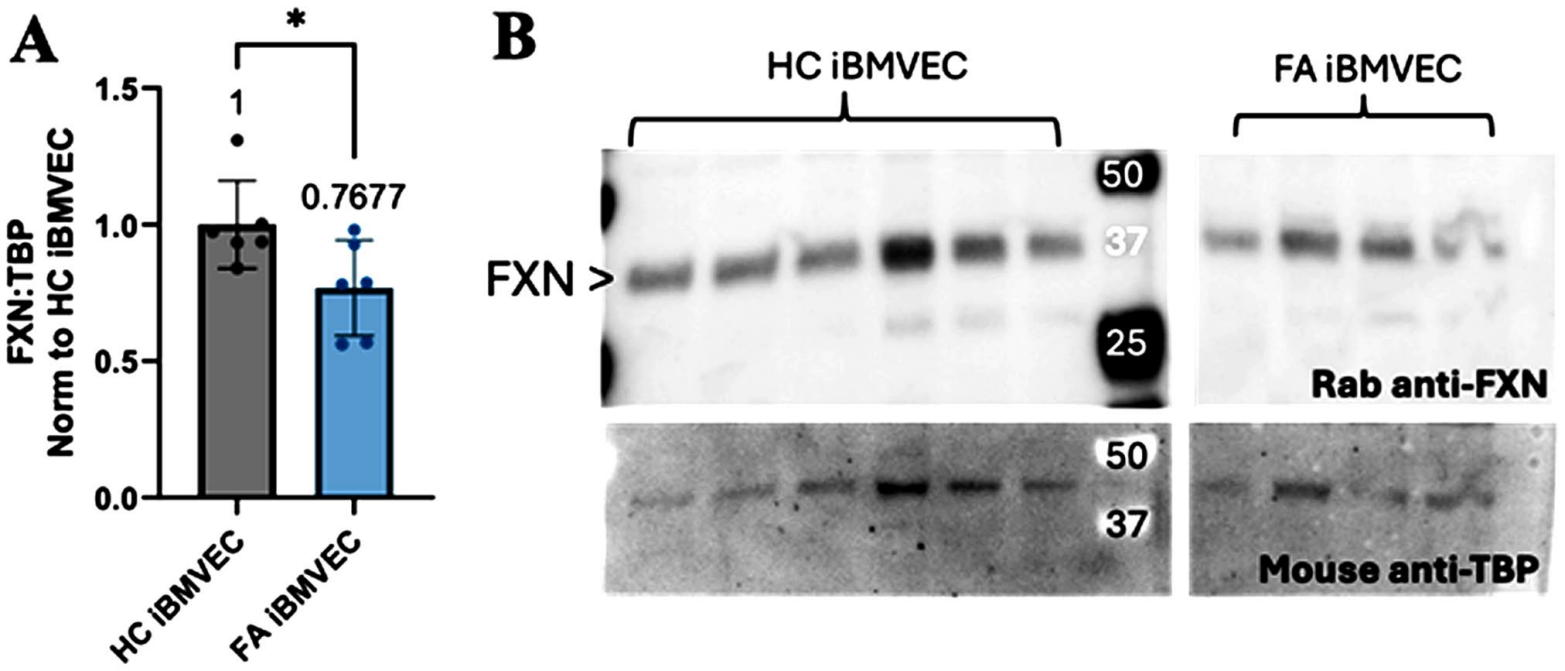
FXN protein expression is decreased in FA iBMVEC. **(A)** Total lysates from HC iBMVEC and FA iBMVEC are blotted for FXN and TATA-binding protein (TBP). Densitometry of the two is used to determine the relative FXN expression level as normalized to HC iBMVEC. The mean of each column is represented below the brackets of significance. **(B)** Representative blot shown, with the major FXN band at ~36 kDa marked with arrowheads. Student’s t-test, α = 0.05. *, *p* < 0.05. Biological replicates: *n* = 6 each.

Frataxin (FXN) protein expression was quantified in the healthy control (HC) and FA iBMVECs using western blotting (Figure 2). FXN undergoes two post-translational cleavage events, resulting in a protein product of 18 kDa (intermediate form) or 14 kDa (mature form) molecular mass (Weng et al., 2019). While we could not visualize a low-molecular-weight band for FXN, a strong band of approximately 36 kDa likely represents a dimerized form of the intermediate protein (Figure 2B). When normalized to the housekeeping gene TATA-binding protein (TBP), FXN protein expression in FA iBMVEC is reduced by approximately 25% (Figure 2A). It is notable that this is a milder downregulation compared to the average FXN patient cell, where FXN protein expression is ~30% that of that in controls (Lazaropoulos et al., 2015). However, this knockdown falls within the range of disease pathology, as patients with late-onset FA retain, on average, approximately 66% of FXN (Sacca et al., 2011).

### FA iBMVEC exhibits decreased oxidative energy metabolism with a shift to glycolysis

**Figure 3:**
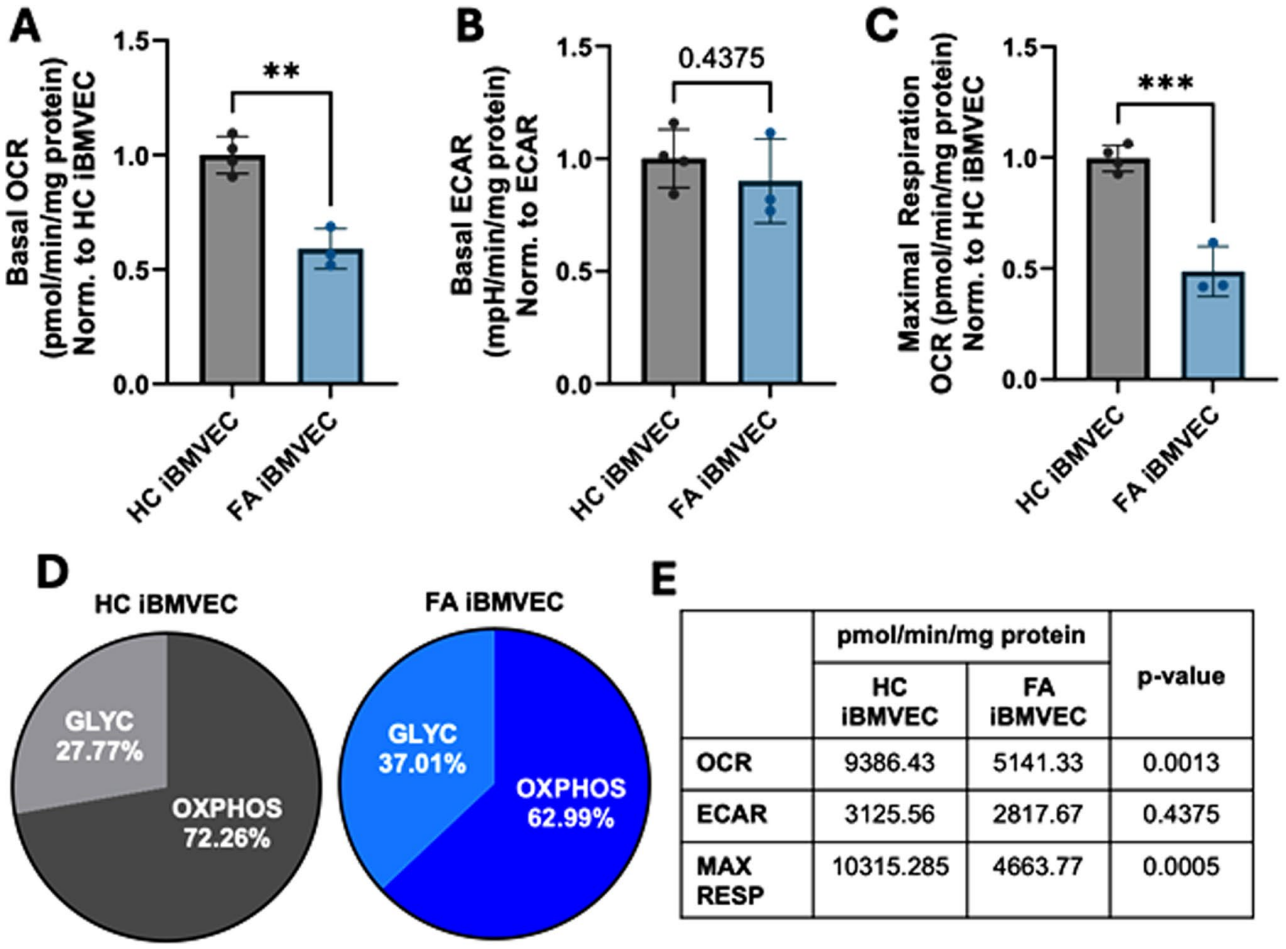
FA iBMVEC has metabolic defects. **(A)** Basal oxygen consumption rate (OCR), **(B)** basal extracellular acidification rate (ECAR), and **(C)** maximal oxidative respiration are measured in the Agilent Seahorse Mito Stress Test, normalized to protein content in the well and further represented as a ratio to HC iBMVEC. **(D)** The ratio of oxidative stress (OXPHOS) and glycolysis (GLYC) per total energy metabolism. **(E)** Numeric values for respiratory measures are represented in panels **(A-C)**. Student’s t-test, α = 0.05. **, *p* < 0.01; ***, *p* < 0.001. Biological replicates: *n* = 4 (HC) and 3 (FA).

Decreased oxidative energy metabolism is a hallmark of FA pathology due to the reliance of the electron transport chain on iron–sulfur clusters. Thus, we sought to quantify oxidative and non-oxidative energy metabolism pathways in iBMVEC using the Agilent Seahorse mito stress test (Lynch and Farmer, 2021). In line with classical FA pathology, FA iBMVEC exhibit a reduced oxygen consumption rate (OCR) compared to HC iBMVEC, exhibiting ~55% of the control’s oxidative energy metabolism (Figure 3A). We also noticed a ~10% decrease in the extracellular acidification rate (ECAR); however, this increase in glycolysis was not statistically significant (Figure 3B). Importantly, total maximal oxidative respiration is reduced by 50% in FA iBMVEC compared to HC iBMVEC (Figure 3C).

Despite the downregulation of both oxidative and non-oxidative energy metabolism, FA cells shift to glycolytic energy production, evidenced by a ~10% increase (Figure 3D). This shift is likely due to the reliance of ETC complexes I-IV on iron prosthetic groups for electron flux. Additionally, the lack of NAD^+^ production during oxidative phosphorylation, coupled with a more oxidative redox environment, probably limits glycolytic capacity as well. In total, we observed decreased oxidative energy metabolism, maximal respiration, total energy metabolism, and glycolysis, with a shift towards anaerobic metabolism in FA iBMVEC. These are all cellular phenotypes characteristic of FA pathology (Lynch and Farmer, 2021).

### FA iBMVEC displays an iron starvation phenotype

**Figure 4:**
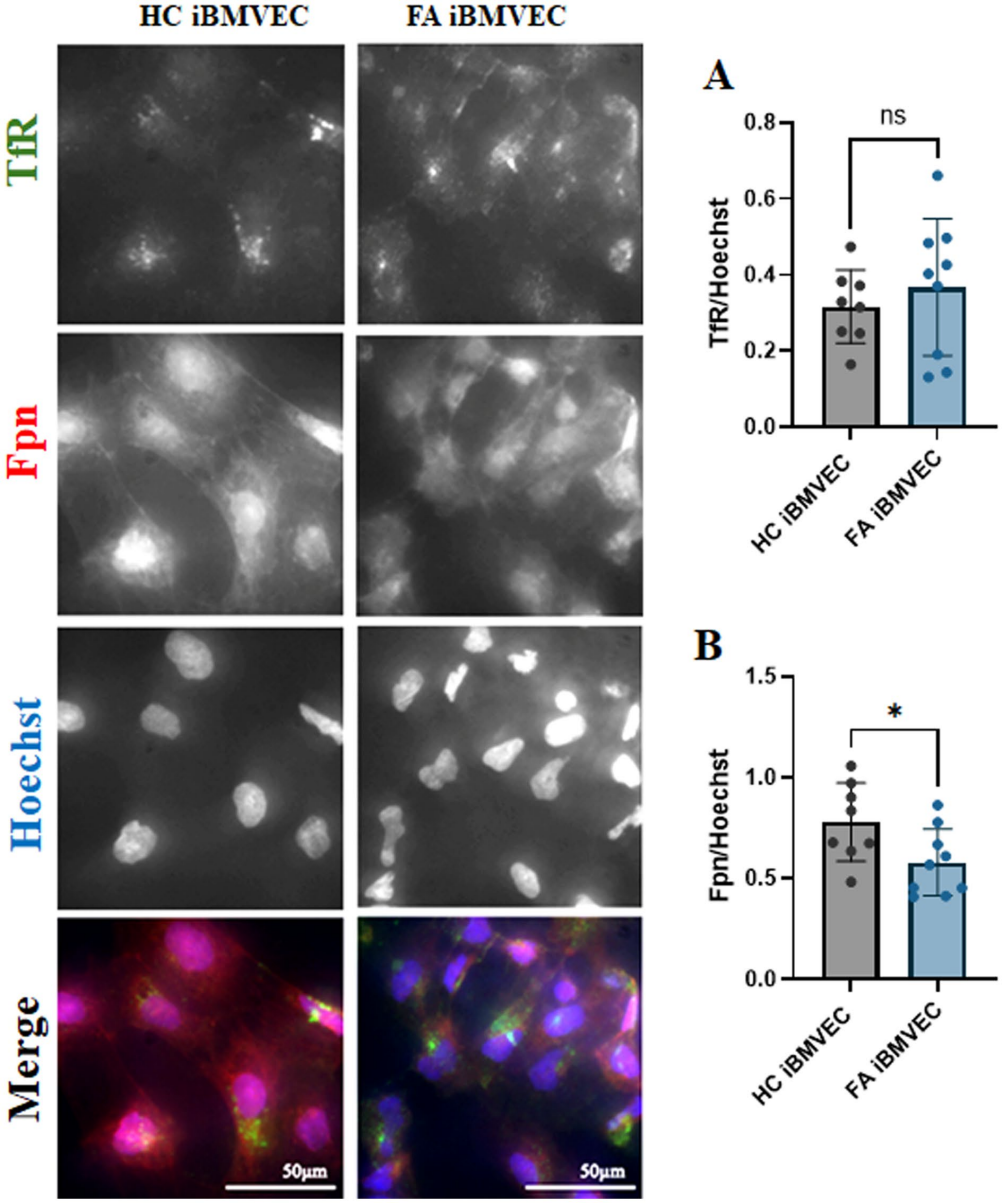
FA iBMVEC displays the known iron starvation phenotype. Healthy control (left) and FA (right) iBMVEC are fixed and stained for transferrin receptor (TfR, green), ferroportin (Fpn, red), and Hoechst. **(A)** Total TfR staining normalized to nuclear stain. **(B)** Total Fpn staining normalized to nuclear stain. All images were taken at 40X magnification. Student’s t-test, α = 0.05. *, *p* < 0.05; ns; non-significant. Biological replicates: 2 (HC) and 3 (FA) coverslips, *n* = 8 (HC) and 9 (FA).

FA cells can be considered iron-starved due to inefficient iron chaperoning, leading to a perceived intracellular iron deficit. This condition prompts the cells to increase iron uptake through the transferrin receptor (TfR) and decrease iron export via ferroportin (Fpn) (Llorens et al., 2019). We examined the expression of both TfR and Fpn in iBMVEC using indirect immunofluorescence. The expression of TfR increased slightly to 116% of HC iBMVEC (Figure 4A), while Fpn decreased significantly to 74% of HC iBMVEC (Figure 4B). Although the changes in TfR are smaller than those in Fpn, the data support the conclusion that FA iBMVEC are sequestering more iron intracellularly, as Fpn is the only known iron exporter.

### FA iBMVEC have intracellular and intra-mitochondrial iron accumulation

**Figure 5:**
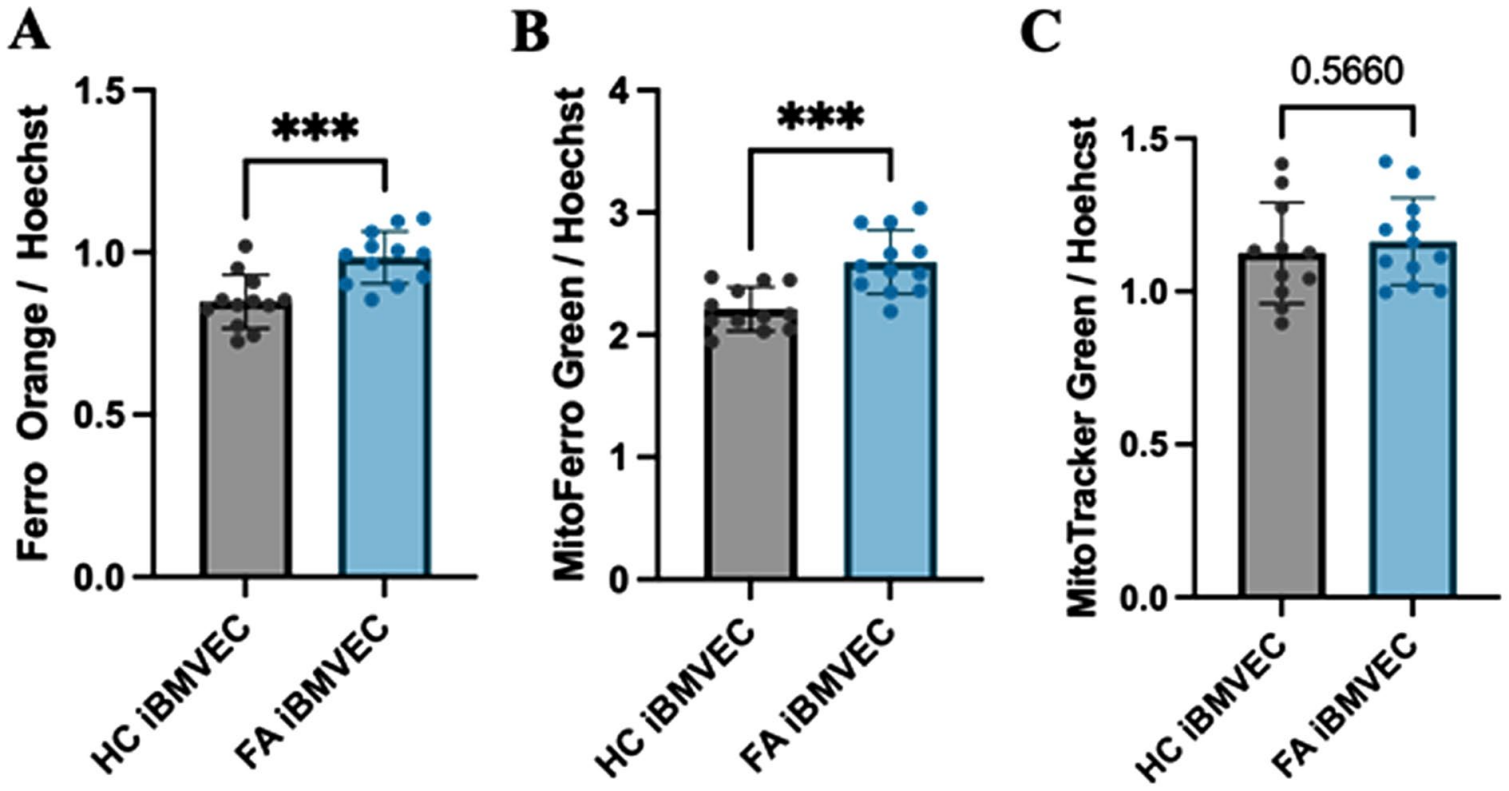
FA iBMVEC has increased cytosolic and mitochondrial labile iron. iBMVEC were stained with **(A)** FerroOrange, **(B)** MitoFerro Green, and **(C)** MitoTracker Green, along with Hoechst-33342. Spectrofluorometry was used to quantify the intensity of each stain as normalized to Hoechst. Student’s t-test, α = 0.05. ***, *p* < 0.001. Biological replicates: **(A,B)** *n* = 12 each and **(C)** *n* = 11 (HC) and 12 (FA).

Intra-mitochondrial and cytosolic iron accumulation are hallmarks of FA pathophysiology (Pandolfo and Hausmann, 2013). Therefore, we quantified the labile ferrous iron in FA iBMVEC. The fluorescent dyes Ferro Orange and MitoFerro Green detected cytosolic and mitochondrial labile iron, respectively (Figures 5A,B). Both dyes indicated statistically significant increases in iron levels in the FA iBMVEC, with 116% enrichment in the cytosolic iron and 122% in the mitochondrial iron compared to controls. Importantly, the increase in the mitochondrial iron pool of FA iBMVEC was not due to an increase in mitochondrial numbers, as detected by MitoTracker Green (Figure 5C).

### FA iBMVEC has decreased Nrf2 immunofluorescent staining

**Figure 6:**
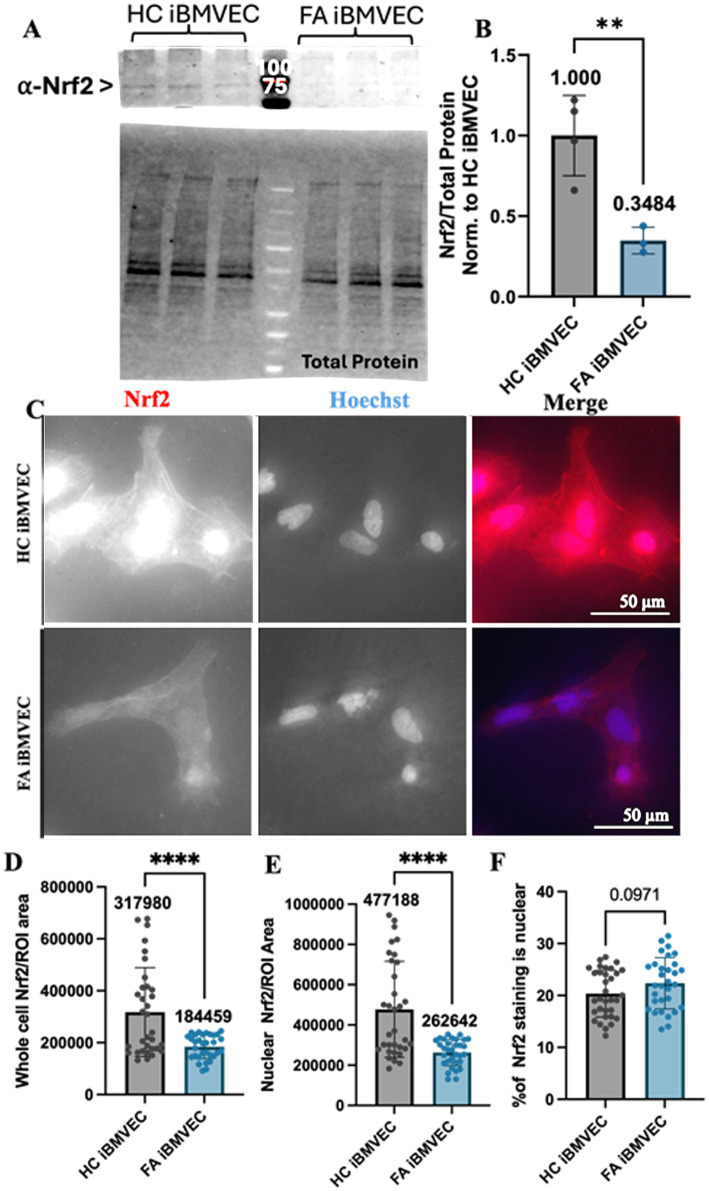
Nrf2 expression, but not nuclear localization, is decreased in FA iBMVEC. **(A,B)** iBMVEC lysates were electrophoresed on a stain-free gel, transferred to PVDF, and probed for Nrf2 as normalized against total protein. **(C–F)** Healthy control and FA iBMVEC were fixed and stained for Nrf2 (red) and Hoechst. **(D)** Regions of interest (ROI) were drawn around 2–5 individual cells per image. Total cell Nrf2 was quantified and normalized to the area of the ROI drawn. **(E)** ROI was drawn around the nuclei from the same cells quantified in **(D)**, and the staining intensity of Nrf2 was normalized to the area of the ROI drawn. **(F)** The staining intensity of nuclei is divided by that of its cell multiplied by 100. All images were taken at 40X magnification. Student’s t-test, α = 0.05. **, *p* < 0.001, ****, *p* < 0.0001. Biological replicates: **(B)** *n* = 3; **(C)** 2 (HC) and 3 (FA) coverslips; cells quantified: **(D,E)** *n* = 33 (HC) and 32 (FA), **(F)** *n* = 33 (HC) and 31 (FA).

Oxidative stress due to decreased iron chaperoning in FA pathology is exacerbated by a lack of Nrf2 activity (Paupe et al., 2009). Indeed, decreases in Nrf2 expression, nuclear localization, and/or activity are hallmarks of many FA model systems and patients (Paupe et al., 2009; Petrillo et al., 2019). Total Nrf2 expression was quantified using western blotting, revealing less than 35% residual protein expression in FA iBMVEC (Figures 6A,B). Despite higher-than-average FXN protein retention in FA iBMVEC compared to average FA patients (Figure 2A), this still indicates a significant reduction in Nrf2.

We also aimed to quantify the amount of nuclear, and thus active, Nrf2 through indirect immunofluorescence. Regions of interest (ROI) were drawn around 2–5 individual cells per image for each condition, and the level of cellular Nrf2 staining was normalized to the area of the ROI (Figures 6C,D). Another ROI was established around the nucleus of the same cells, with the staining intensity normalized to this area (Figure 6E). The proportion of nuclear Nrf2 staining (Figure 6F) was quantified by normalizing the nuclear staining to the whole-cell staining and converting it into a percentage. Based on indirect immunofluorescence, we found a significant decrease in total Nrf2 protein expression to approximately 58% of the HC iBMVEC controls (Figures 6C,D). This finding was also supported by the relative intensity of nuclear Nrf2 staining, with FA iBMVEC exhibiting 55% of that observed in the HC iBMVEC (Figure 6E). This proportion does not correlate with the varying levels of Nrf2 co-localizing with the nuclear compartment (Figure 6F). This suggests that FXN deficiency in this model leads to reduced Nrf2 production but does not affect Nrf2 nuclear translocation.

### FA iBMVEC have disproportionate actin staining at peripheral membranes

**Figure 7:**
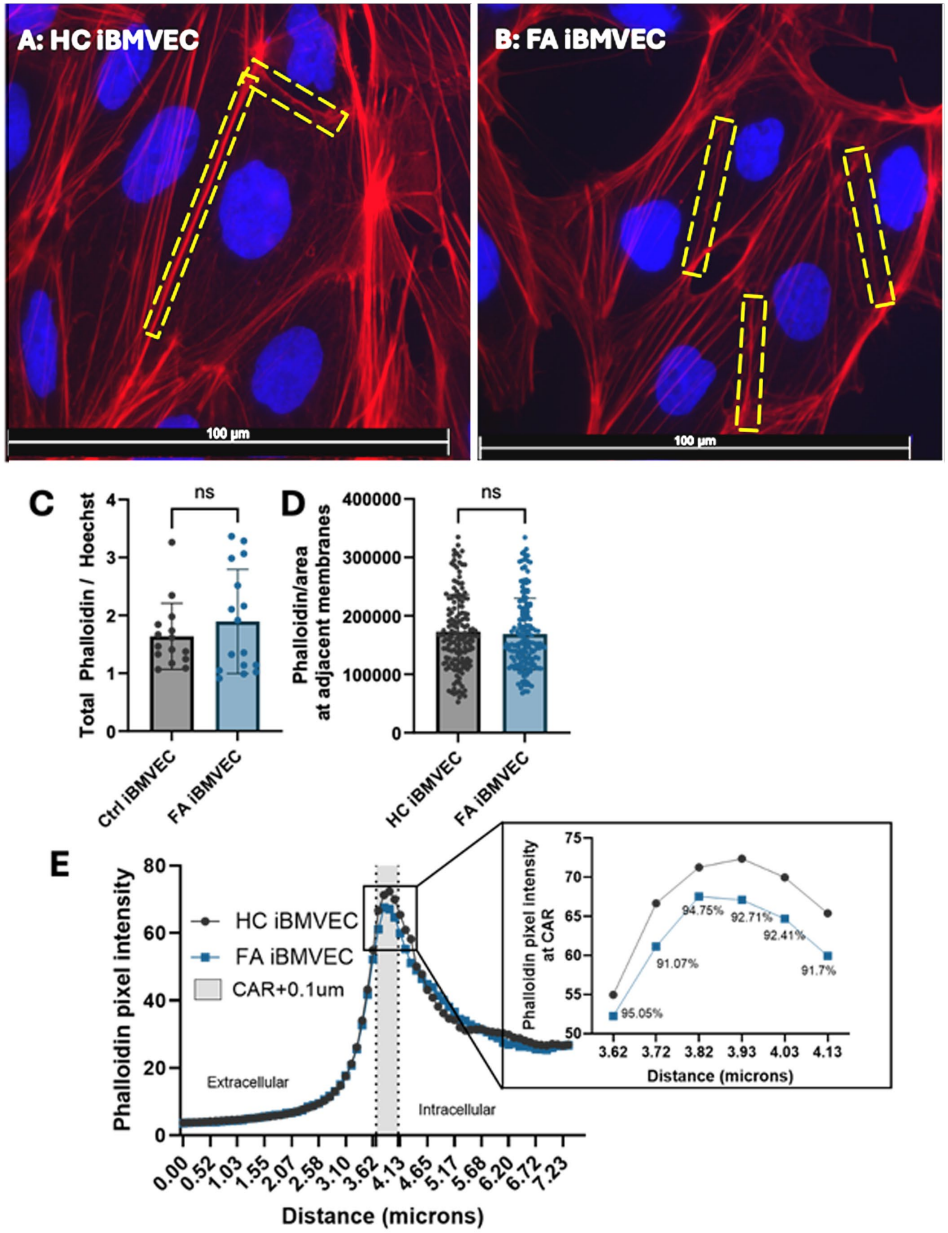
FA iBMVEC has relatively normal F-actin abundance. **(A)** Healthy control and **(B)** FA iBMVEC are fixed and incubated with Phalloidin Texas Red-X and Hoechst. **(C)** Total filamentous actin staining normalized to nuclear stain. **(D)** Regions of interest are drawn around bicellular adjacent cell membranes, and pixel intensity is normalized to the area of the ROI drawn. **(E)** A 7.34 μm line was drawn through 1–2 non-touching plasma membranes (PM) of cells in the field of view, and histograms of pixel intensity were averaged. 300 nm inside the cell, described as the CAR (+0.1 μm accounting for error), is denoted between the dotted lines and shaded gray. The inset shows the data points in the CAR with the corresponding % of FA F-actin at each point compared to HC. All images were taken at 63X magnification **(C, D)** Student’s t-test, α = 0.05. Biological replicates: 3 (HC) and 6 (FA) coverslips. Images analyzed: **(C)** *n* = 15 (HC) and 16 (FA). Cell membranes analyzed: **(D)** *n* = 156 (HC) and 163 (FA). **(E)** *n* = 173 (HC) and 175 (FA).

Increased actin glutathionylation was initially identified in fibroblasts from FA patients. This post-translational modification occludes the actin-actin binding sites of monomers, decreasing both the rate and extent of F-actin formation as well as filament stability (Pastore et al., 2003; Piemonte et al., 2001; Dalle-Donne et al., 2003; Paupe et al., 2009). We examined the abundance of F-actin using Phalloidin-Texas Red, a dye that binds to actin-actin interfaces, thereby quantifying only filamentous actin (Figures 7A,B). This was disrupted in our previous model of shFXN immortalized BMVEC at the whole-cell, membrane, and CAR structures, the former being important for tethering transmembrane proteins like those found in TJ complexes (Smith and Kosman, 2024).

Interestingly, FA iBMVEC did not display any statistically significant changes in F-actin abundance or organization. Although FA iBMVEC exhibited increased F-actin staining at the whole-cell level, this effect was not statistically significant (Figure 7C). To quantify the F-actin at adjacent cell membranes, a region of interest was drawn around the interface of two neighboring cells, and the pixel intensity was normalized to the defined area. This also resulted in an insignificant change: FA iBMVEC had approximately 3% less F-actin at the bi-cellular membranes (Figure 7D).

To quantify the amount of F-actin at peripheral membranes, the outer membranes of cells (not attached to adjacent cells) were bisected with a line measuring 7.44 mm, and the intensity of actin staining was measured (Figure 7E) (Dagda et al., 2009; Steimle et al., 2022; Smith and Kosman, 2024). The midpoint of the line was designated at the beginning of the cell membrane, with the left segment representing the extracellular space and the right segment representing the intracellular space. The lines were averaged and graphed, with a dotted line marking the start of the peripheral membrane (3.655 mm) and another dotted line at 40.65 mm. The shaded region in between represents the CAR, a 300 mm structure beneath the plasma membrane that is important for anchoring the cytosolic tails of transmembrane proteins (Belvitch et al., 2018). An additional 100 nm was added to the CAR to account for any potential error in line placement. The CAR is enlarged in the inset, with values below indicating the percent change between HC and FA iBMVEC (Figure 7E, inset). FA iBMVEC exhibit a mild deficiency in F-actin organization in the CAR (~6–9% loss), although these changes were not statistically significant.

Although these differences were not significant, a dichotomy exists between total actin staining and membranous organization in FA iBMVEC; a ~16% increase in total actin staining was observed, contrasting with a ~10% decrease in CAR organization (Figures 7A,C). This pattern suggests an altered organization of F-actin around the FA cell.

### FA iBMVEC do not have increased actin glutathionylation

**Figure 8:**
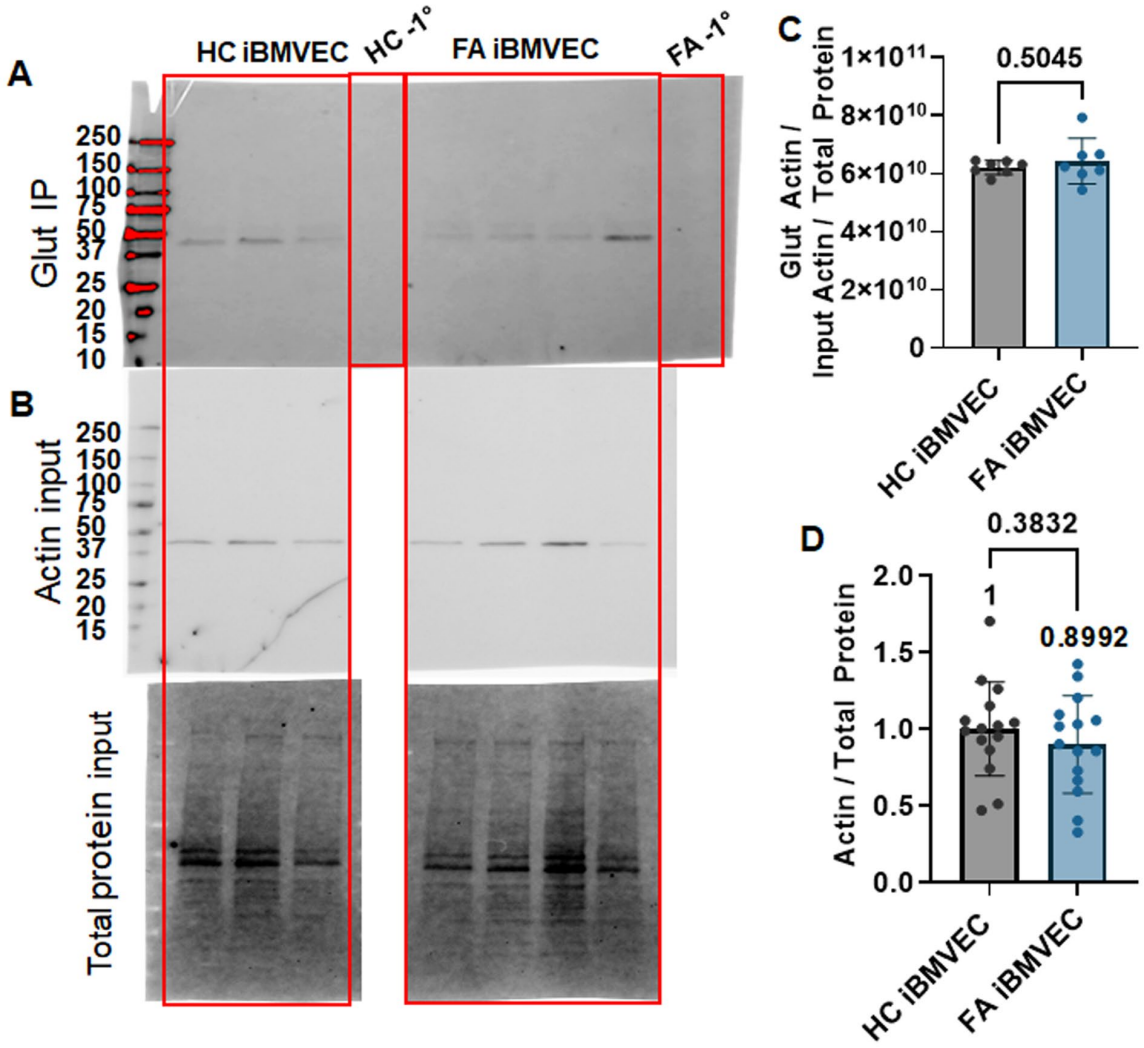
FA iBMVEC does not have increased actin glutathionylation. HC and FA iBMVEC lysates (150 μg) were pre-cleared and incubated with α-glutathione A/G agarose beads. One control each included A/G beads in the absence of the α-glutathione primary to assess non-specific binding (−1°). **(A)** Reactions were electrophoresed on a 4–20% Stain-Free gel and probed for *β*-actin. **(B)** Non-IP lysates (15 μg) were probed for β-actin as normalized to total protein transferred (input blot). **(C)** Densitometry is used to quantify the intensity of the IP band per the normalized actin input band. **(D)** Actin per total protein. Student’s t-test, α = 0.05. Biological replicates: **(C)** *n* = 7; **(D)** *n* = 15.

Using GSSP-specific immunoprecipitation, we quantified actin glutathionylation as an alternative means to examine actin dynamics in FA iBMVEC. HC and FA iBMVEC lysates were pre-cleared to reduce non-specific binding with protein A/G agarose beads and then incubated with beads coated in mouse anti-glutathione. Each HC and FA iBMVEC lysate sample underwent incubation with beads in the absence of a primary antibody (−1°) to assess non-specific binding. Lysates were subjected to electrophoresis on a stain-free gel and transferred to PVDF, where they were probed for precipitating actin using rabbit anti-β-actin (Figure 8A). The lysate that was not incubated with either beads or primary antibody was electrophoresed and probed for actin under the same conditions to represent the “input” samples (Figure 8B). In this analysis, the FA iBMVEC sample showed no increase in actin glutathionylation (Figure 8C), consistent with the lack of change in the abundance of F-actin (Figure 7).

### FA iBMVEC are deficient in Claudin-5, ZO-1, and Occludin protein expression

**Figure 9:**
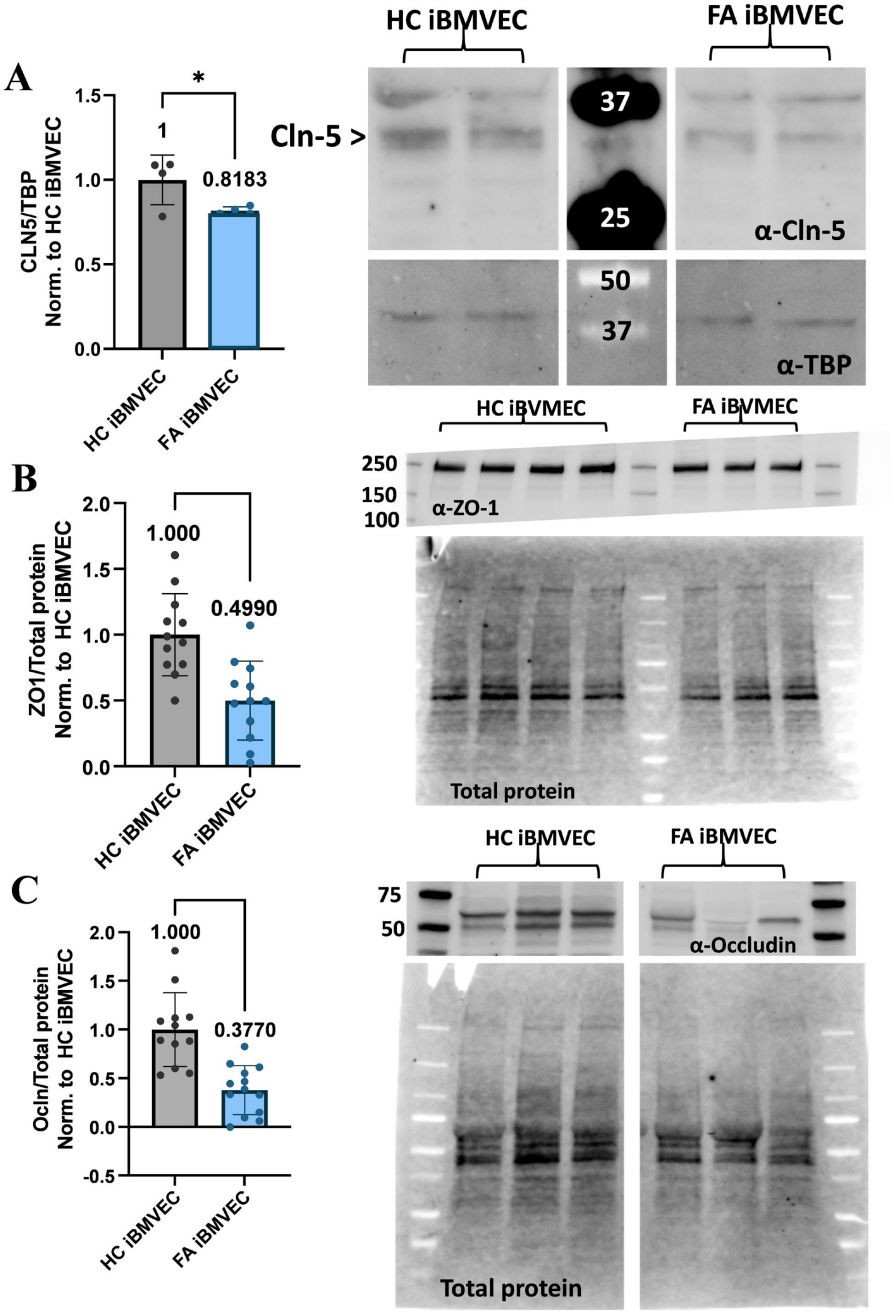
FA iBMVEC is deficient in Claudin-5, Occludin, and ZO1 tight junction proteins. **(A)** iBMVEC lysates were electrophoresed, transferred to nitrocellulose, and probed for Claudin-5 (Cln5) and TBP. The major band for Claudin-5 is represented by the arrowhead. Bands are quantified by densitometry, normalized to TBP as the housekeeping gene, and further normalized to HC iBMVEC. Fold change is written above the bars. **(B,C)** iBMVEC lysates were electrophoresed on a stain-free gel and transferred to PVDF. Total protein activation is used for normalization. Blots are probed for **(B)** ZO-1 and **(C)** Occludin (Ocln) were quantified using densitometry, normalized to total protein content per lane, and further normalized to HC iBMVEC. Representative blots are shown. Student’s T-test, α = 0.05. *, *p* < 0.05; ***, *p* < 0.001; and ****, *p* < 0.0001. Biological replicates: **(A)** *n* = 4; **(B)** *n* = 4; **(C)** *n* = 12 (HC) and 13 (FA).

We sought to quantify the abundance of TJ proteins, which were found to have decreased at both the transcriptional and translational levels in our previous shFXN immortalized BMVEC model (Smith and Kosman, 2024). Western blotting was used to quantify the protein expression of the TJ proteins Claudin-5 (Figure 9A), ZO-1 (Figure 9B), and Occludin (Figure 9C). Relative to either the housekeeping gene TBP (for Claudin-5, Figure 9A) or total protein staining (for ZO-1 and Occludin, Figures 9B,C), the FA iBMVEC displayed reduced protein expression of all three TJ proteins. FA iBMVEC expressed approximately 80% of Claudin-5, 50% of ZO-1, and 37% of Occludin. These observations, along with those reported previously (Smith and Kosman, 2024), indicate that the reductions in TJ protein abundance are an inherent phenotype of FA, following the decline in FXN expression. These changes in TJ protein levels likely have implications for barrier physiology, a premise not previously investigated in disease. As demonstrated below, the barrier properties of FA iBMVEC are notably impaired.

### FA iBMVEC have decreased intercellular Claudin-5 and Occludin staining but retain intercellular ZO-1 staining

**Figure 10:**
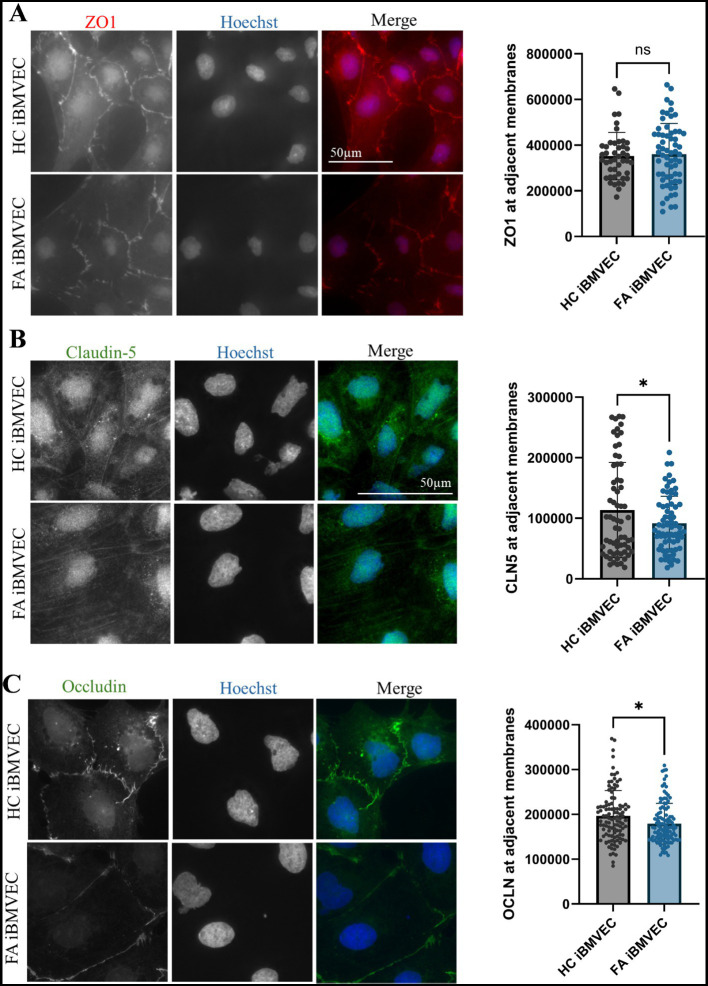
FA iBMVEC has decreased intercellular staining of Claudin-5 and Occludin, but not ZO-1, compared to HC iBMVEC. iBMVEC were fixed and stained for **(A)** ZO-1, **(B)** Claudin-5, and **(C)** Occludin and Hoechst. Regions of interest were drawn around bi-cellular adjacent cell membranes, and pixel intensity was normalized to the area of the ROI drawn. Student’s T-test, α = 0.05. ns; non-significant. Biological replicates: 2 (HC) and 3 (FA) coverslips; membranes quantified: **(A)** *n* = 65 (HC) and *n* = 73 (FA), **(B)** membranes quantified: *n* = 107 (HC) and 114 (FA), **(C)** membranes quantified: *n* = 45 (HC) and 62 (FA).

Also key to barrier function is the precise architecture of the TJ proteins. Thus, we aimed to quantify the abundance of TJ staining at bi-cellular/intercellular interfaces using indirect immunofluorescence, similar to the imaging of F-actin. We identified regions of interest at adjacent cell membranes based on nuclear juxtaposition and quantified pixel intensity in relation to ROI area (Figure 7D). For the cytosolic TJ scaffolding protein ZO-1, bicellular examination failed to show any deficit in intercellular organization compared to HC iBMVEC (Figure 10A). This contrasts with the significant decrease in protein expression noted via western blotting (Figure 9B), but may indicate that ZO-1 is organizing at adherens junctions, a phenomenon observed during cell stress (Katsuno et al., 2008). Nonetheless, reductions in transmembrane protein TJ expression and organization are likely to negatively impact barrier function (Naser et al., 2022).

Claudin-5 is the primary regulator of BBB impermeability and is the main claudin isoform examined in our studies (Greene et al., 2019). In line with decreased total Claudin-5 protein expression (Figure 9A), FA iBMVEC shows a statistically significant decrease in bi-cellular staining to ~80% of that in HC iBMVEC (Figure 10B). Similar to Claudin-5, bicellular organization of Occludin was also decreased in FA iBMVEC (Figure 10C), showing a mild yet significant reduction of ~10% compared to HC iBMVEC. Overall, there is a statistically significant reduction in the protein expression of all three TJ proteins (Figures 9A-C) and a statistically significant decrease in bicellular staining of both transmembrane tight junction proteins examined, Claudin-5 (Figure 10B) and Occludin (Figure 10C).

### FA iBMVEC are paracellularly permeable

**Figure 11:**
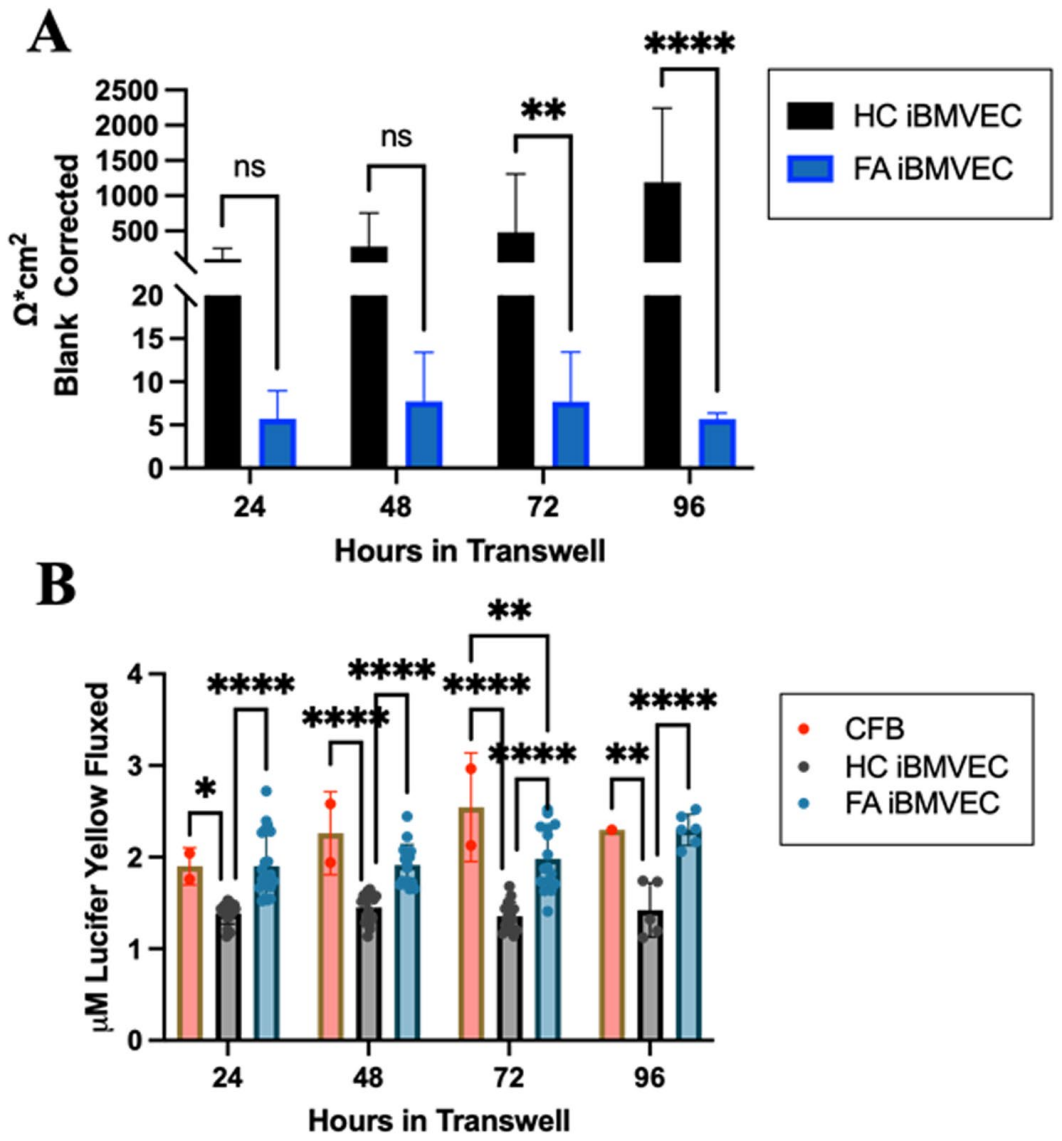
FA iBMVEC is paracellularly permeable compared to HC iBMVEC. **(A)** iBMVEC are plated on collagen and fibronectin-coated transwells, and TEER is quantified at 24-h intervals starting from 24-h post-plating to 96-h. A cell-free blank (CFB) is used to blank-correct at each time point. **(B)** Following TEER, Lucifer Yellow (LY, 50 uM) was added to the apical chamber of transwells and incubated for 45 min. Basal aliquots were measured for flux. Two-way ANOVA, α = 0.05. *, *p* < 0.05; **, *p* < 0.01; ****, *p* < 0.0001; ns; non-significant. Biological replicates: **(A)** 24 h; *n* = 24 each, 48 and 72 h; *n* = 18 each, 96 h; *n* = 6 (HC) and 7 (FA). **(B)** 24 h; *n* = 22 (HC and FA) and *n* = 2 (CFB), 48 h; *n* = 16 (HC), *n* = 17 (FA), and *n* = 2 (CFB), 72 h; *n* = 16 (HC and FA) and *n* = 2 (CFB), 96 h; *n* = 5 (HC), *n* = 6 (FA), and *n* = 1 (CFB).

To investigate barrier formation, iBMVEC were seeded on transwell membranes. Transendothelial electrical resistance (TEER) was measured every 24 h for 96 h starting 24 h post-plating, to assess barrier integrity. A cell-free blank was included to subtract background noise from each sample. The observed resistance values indicate a very strong phenotype of paracellular permeability in the FA iBMVEC compared to HC controls (Figure 11A). The FA iBMVEC barrier starts at less than 6% of the HC iBMVEC at the 24-h timepoint. Additionally, while the resistance values increase over time in the HC iBMVEC due to TJ maturation (Breslin and Yuan, 2024), the FA iBMVEC plateaus at a low resistance value. Consequently, the FA iBMVEC barrier integrity remains less than 1% of HC iBMVEC at 96 h (Figure 11A). This indicates a defect not only in TJ protein expression (Figure 9) and organization (Figure 10) but also in normal barrier maturation.

Following each TEER analysis, 50 μM Lucifer Yellow (LY) was added apically to the cell culture, and its flux into the basal chamber was analyzed. Mammalian cells lack the transport machinery for LY, so the flux between chambers indicates only paracellular permeability. In line with decreased TEER values, FA iBMVEC exhibited significantly more LY flux than HC iBMVEC at all time points examined (Figure 11B). Initially, there was 37% more flux in the FA iBMVEC compared to HC controls at 24 h which progressed to 61% more flux by 96 h. Notably, the LY flux patterns in FA iBMVEC closely resemble those of the cell-free blank (CFB), indicating that these patient-derived cells are significantly hyperpermeable and lack the TJ maturation patterns characteristic of barrier-forming cells.

To validate that the observed permeability in FA iBMVEC is not due to poor coverage on the transwell membrane, HC and FA iBMVEC were seeded on transwells, fixed, and stained with Hoechst-33342 every 24 h to replicate the above permeability experiment. Images of the coverslips were quantified for Hoechst staining intensity, which demonstrated there was no deficit in cell coverage of the FA iBMVEC (Supp. Figure 2). Thus, this finding confirms that FA iBMVEC is paracellularly permeable independent of changes in cell proliferation or death or differential seeding patterns. Combined with decreases in TJ protein expression and organization, we conclude that FA iBMVEC forms inherently poor barrier systems. This novel finding should be considered relevant to other barrier-forming tissues and as a distinct feature of disease pathology.

## Discussion

This study sought to investigate barrier physiology in an *in vitro* brain microvascular endothelial cell (BMVEC) model of FA as a follow-up manuscript to our previous publication indicating changes in tight junction protein expression and barrier disruption in shRNA-mediated FXN knockdown in immortalized BMVEC (Smith and Kosman, 2024). The latter model indicates the direct downstream effects of post-transcriptional FXN knockdown, while our current studies have the added element of potential genetic manipulations consequent to the GAA expansion repeat, such as the cis-silencing of the flanking gene *PIP5K1B* (Bayot et al., 2013). Interestingly, in each model, we found inherent decreases in tight junction protein expression and subsequent barrier integrity (Figures 8–11; Smith and Kosman, 2024). The exacerbation of the phenotype observed here *compared to* our immortalized cell model may indicate synergistic negative genetic interactions resulting from the expansion repeat, which were absent in our previous studies. Thus, our current report aims to elucidate new molecular pathways disrupted in the disease and advance the investigation of vascular barriers in FA.

We acknowledge the limitations of the current studies, which have a biologic n value of one for each control and FA patient iPSC. Our novel proof-of-concept findings would benefit from additional research using various available cell lines, such as those from the Friedreich’s Ataxia Cell Line Repository (FACLR) (Li et al., 2016). In this study, we chose to investigate specific molecular pathways affected by the disease to inform future therapeutic exploration rather than focus on achieving higher statistical significance with a pooled patient sample group. Continued investigation of this premise would enhance the understanding of the molecular pathways of the disease and the penetrance of this phenotype in the patient population.

The FA iPSCs were a donation from a 34-year-old male patient who was homozygous for the GAA expansion. Despite being near the average life expectancy of an FA patient at the time of collection, the residual FXN expression quantified for FA iBMVEC compared to HC iBMVEC was approximately 76% (Figure 2A). This is relatively high for FA patients, where average protein retention ranges from approximately 15 to 30%, depending on the cell type (Lazaropoulos et al., 2015). Such protein retention is not unheard of, as in late-onset FA, where patients are diagnosed after age 25 and retain, on average, approximately 66% of FXN expression (Bhidayasiri et al., 2005; Fearon et al., 2020; Sacca et al., 2011). Late-onset FA patients comprise approximately 25% of the total FA population and often present with milder symptoms (Fearon et al., 2020).

Significantly, our system with ~25% reduced FXN expression shows a large defect in barrier integrity in FA iBMVEC (Figure 11). This finding also applies to our shFXN immortalized hBMVEC, indicating that FA BMVEC is inherently deficient in normal tight junction and barrier functions (Smith and Kosman, 2024). Our findings highlight the importance of investigating non-canonical disease pathologies and tissue systems to enhance molecular understanding of FA.

Our FA iBMVEC model replicates key characteristics of FA, including metabolic defects. The FA iBMVEC exhibits a reduced rate of mitochondrial metabolism and a shift towards glycolysis (Figures 3A,D). Glycolysis is also slightly decreased (Figure 3B). In FA, the main metabolic defect lies in the function of the electron transport chain, which likely reduces glycolytic capacity due to decreased cycling of NAD+/NADH.

The FA iBMVEC replicates the iron starvation hypothesis. FA cells detect the lack of iron–sulfur cluster biosynthesis as a deficit in iron availability, leading to the upregulation of iron import proteins such as TfR and the downregulation of the only known iron exporter, Fpn, to increase intracellular iron levels. Through indirect immunofluorescence, we measured approximately a 16% increase in TfR and a ~26% decrease in Fpn protein expression (Figures 4A,B). This is consistent with increased cytosolic and mitochondrial labile iron, indicating cellular sequestration and potential oxidative stress (Figures 5A,B). Furthermore, since Fpn is the only known iron exporter in BMVEC and is mostly expressed on the basolateral membrane, these data suggest that ongoing iron accumulation in the FA patient brain occurs not through transcellular flux. Instead, our demonstration of FA iBMVEC barrier degradation and aberrant paracellular solute flux suggests the possibility of a microbleed or other small, transient permeability regarding abluminal iron accumulation.

In physiological conditions, an appropriate antioxidant response buffers changes in cellular redox potential. However, in FA iBMVEC, the total expression and nuclear abundance of Nrf2 were decreased (Figures 6B,D,E). Despite these reductions, the percentage of total Nrf2 that localized to the nucleus did not differ from HC iBMVEC (Figure 3F). This indicates that Nrf2 abundance, but not its translocation, is altered downstream of FXN decrease. Although not examined further here, this finding is consistent with either an upregulation of KEAP1 and Nrf2 ubiquitination-proteolysis or a decrease in Nrf2 transcript; the latter being true in our previous shFXN hBMVEC model and several others (Smith and Kosman, 2024; Petrillo et al., 2019; La Rosa et al., 2021).

Our original hypothesis that FA iBMVEC would exhibit degraded junctional properties downstream of F-actin destabilization was based on previous findings where increased oxidative stress in FA patient fibroblasts correlated with actin glutathionylation (Pastore et al., 2003; Piemonte et al., 2001). This post-translational modification was later identified as pathological to the filamentous actin formation rate and extent (Dalle-Donne et al., 2003). We quantified increased intracellular iron loading (Figure 5) and a significant deficit in Nrf2 protein expression (Figures 6B,D), suggesting oxidative stress in this model. However, there was only a small, insignificant change in F-actin abundance, with a ~15% increase in total-cell F-actin (Figure 7C) and a 5–10% reduction at the CAR (Figure 7E). This may represent an altered ratio of cytosolic to membranous F-actin. While actin glutathionylation was reported in FA patient fibroblasts, the FA patient cells examined here failed to show any changes in the level of actin glutathionylation as quantified using western blotting (Figure 8C) (Pastore et al., 2003; Piemonte et al., 2001). This indicates that actin glutathionylation is either not as prevalent in disease pathology as originally assumed or that this disease pathology is heterogeneous among the patient population.

In contrast, we observed a significant reduction in protein expression of the TJ proteins, Occludin (37% of control, Figure 9C) and ZO-1 (50% of control, Figure 9B), along with a milder reduction in Claudin-5 expression (81% of control, Figure 9A). Given these altered expression levels, a decrease in bicellular organization of these TJs was anticipated. This holds true for both Claudin-5 (80% of control, Figure 10B) and Occludin (91% of control, Figure 10C), but not for ZO-1 (Figure 10A). This dichotomy may indicate cellular stress, as cells with diminished tight junction-forming capacity transport ZO-1 to adherens junctions to maintain cellular support (Katsuno et al., 2008). Taken together, these results indicate a significant reduction in TJ protein expression and altered organization at bicellular membranes.

Accordingly, the barrier function, as measured by TEER and paracellular solute flux, is significantly reduced in the FA iBMVEC model (Figure 11). The raw resistance values of the FA iBMVEC, recorded by TEER, remained only a few ohms above a cell-free blank at each time point, from 24 to 96 h in culture. In contrast, the HC iBMVEC surpassed the cell-free blank by over 1,000 ohms at 96 h (Figure 11A). Importantly, the resistance of the HC iBMVEC demonstrates TJ maturation, showing increasing resistance values over time. However, this is not the case for the FA iBMVEC, which exhibits a nearly plateaued resistance value over the same timeline (Figure 11A).

These TEER values correlate with the increased flux of the paracellular solute Lucifer Yellow (LY) (Figure 11B). Two important conclusions can be drawn from these flux data. First, HC iBMVEC plateaus at approximately 1.3 μM LY, despite the maturation of TEER values. TJs are naturally permeable to solutes less than approximately 400 Da (Gawdi and Emmady, 2020). LY, at 521 Da, may therefore flux in small amounts. Secondly, at all time points, the FA iBMVEC significantly fluxes more LY than the HC iBMVEC (approximately 1.9 μM), a value that is not significantly different from the cell-free blank at most time points (~2.2 μM, Figure 11B). Together with TEER data, this suggests that the FA iBMVEC lack continual TJ maturation (Figure 11A) and the steady-state paracellular flux resistance seen in HC iBMVEC (Figure 11B). Overall, the FA iBMVEC exhibit minimal resistance to small ionic solutes detectable via TEER and increased permeability to a 500 Da solute. Further investigation using paracellular tracers of varying molecular weight would provide more insight into the extent of paracellular permeability demonstrated by an FA iBMVEC barrier.

A significant deficit in barrier integrity led to an examination of cell coverage on the transwell membrane, as increased cell death or poor cell attachment would lead to noticeable permeability. However, during the assessment of cell coverage on a transwell membrane using Hoechst staining, we observed no changes in nuclear staining, a surrogate marker for cell density, at any of the time points investigated (Supplementary Figure 2). This indicates that the FA iBMVEC attaches to and colonizes the surface of the transwell membrane like HC cells, yet they are significantly deficient in barrier integrity (Figure 11 and Supplementary Figure 2). Taken together, these data indicate that cells lacking FXN are inherently deficient in TJ protein expression and therefore exhibit inadequate barrier integrity. Importantly, these changes occur independently of alterations in actin glutathionylation or F-actin abundance (Figures 7,8). Thus, the observation that TJ proteins and associated barrier integrity are inherently reduced in FA BMVEC presents a novel insight into the molecular understanding of the disease. These findings may have implications regarding the progressive brain iron accumulation, neuroinflammation, neurodegeneration, and general neurovascular health observed in FA patients. Additionally, as nearly 7% of FA patients experience stroke related to atrial fibrillation or mural thrombosis (Tsou et al., 2011), alterations in BBB integrity may be aggravated by such vascular pressure insults. While this research specifically focuses on the brain microvasculature, further studies are needed to explore other key barrier systems to fully understand the whole-body manifestations of FA.

## Conclusion

The post-translational modification of actin glutathionylation has been known as a consequence of oxidative stress in FA fibroblasts for over 20 years, yet the resulting effects on cytoskeletal pathology and cellular architecture remain unknown. Additionally, the FA literature has only investigated barrier systems in the past 5 years. Here, we identified that FA patient-derived iPSCs differentiated into brain microvascular endothelial-like cells exhibit inherent deficits in tight junction protein expression, consistent with barrier hyperpermeability. These phenotypes are independent of changes in F-actin abundance. This implies that FA barriers are inherently compromised, regardless of changes to cytoskeletal organization. Investigating this premise has implications for the homeostasis of the patient’s neurovascular unit and should be extrapolated to other barrier systems in the disease or informed in the investigation of therapeutic interventions. In the context of the brain microvasculature, targeting such pathologies may alleviate the progressive brain iron accumulation, neurodegeneration, neuroinflammation, and overall neurovascular health challenges faced by FA patients. In summary, aberrant barrier physiology is an overlooked issue in FA, which could represent new druggable targets to alleviate patient symptoms.

## Data Availability

The original contributions presented in the study are included in the article/Supplementary material. Further inquiries can be directed to the corresponding author.
