# Supplementary material for: Brain microvascular endothelial cells differentiated from a Friedreich’s Ataxia patient iPSC are deficient in tight junction protein expression and paracellularly permeable
Source: Front Mol Neurosci. 2025 Apr 15;18:1511388. doi: 10.3389/fnmol.2025.1511388 (PMC12037585; doi:10.3389/fnmol.2025.1511388)
Supplement: Supplementary file 1 [file Data_Sheet_1.docx]

Brain microvascular endothelial cells differentiated from a Friedreich’s Ataxia patient iPSC are deficient in tight junction protein expression and paracellularly permeable

Frances M. Smith^1^ and Daniel J. Kosman^1*^

Supplemental Materials

**Supplemental Materials and Methods**

**Western Blotting: Differentiation Markers**

iBMVEC lysates were prepared as previously described, and electrophoresed on a 4-20% Bis-Tris Gel (ThermoFisher). Proteins were transferred to PVDF as previously described. Blots were probed for the BMVEC differentiation markers GLUT-1 and PECAM-1 [24]. All bands were quantified using densitometry and normalized to the housekeeping control TBP.

**Supplemental Tables**

Supplemental Table 1. Antibodies used in western blotting.

| **Primary Antibodies – Western Blotting** | | | |
| --- | --- | --- | --- |
| *Target* | *Catalog number* | *Concentration* | *Secondary detection* |
| Beta-Actin | Cell Signaling Technologies  #8457 | 1:5,000 | Donkey α-rabbit: Alexa Fluor 647 |
| Claudin-5 | Abcam #ab131259 | 1:1,000 | Goat α-rabbit: HRP |
| Frataxin | ThermoFisher # PA5-13411 | 1:500 | Goat α-rabbit: HRP |
| GLUT-1 | Abclonal #A6982 | 1:1,000 | Goat α-rabbit: HRP |
| Nrf2 | Abclonal #A21176 | 1:1,000 | Donkey α-rabbit: Alexa Fluor 647 |
| Occludin | Abclonal #A2601 | 1:1,000 | Donkey α-rabbit: Alexa Fluor 647 |
| PECAM-1 | Abclonal #A0378 | 1:1,000 | Donkey α-rabbit: Alexa Fluor 647 |
| TATA-binding protein | ThermoFisher #49-1036 | 1:2,500 | Donkey α-mouse: Alexa Fluor 488 |
| ZO-1 | Abclonal #A0659 | 1:5,000 | Donkey α-rabbit: Alexa Fluor 647 |
| **Secondary Antibodies – Western Blotting** | | | |
| *Target* | *Catalog Number* | *Concentration* | |
| Donkey α-rabbit: Alex Fluor 647 | ThermoFisher #A-31573 | 1:1,000 | |
| Donkey α-mouse: Alexa Fluor 488 | ThermoFisher #A32766TR | 1:5,000 | |
| Goat α-rabbit: HRP | Cell Signaling Technologies #7074 | 1:5,000 | |

**Supplemental Table 2. Antibodies used in immunofluorescence.**

All antibodies were used at a 1:1,000 dilution.

| **Primary antibodies – Indirect Immunofluorescence** | | | |
| --- | --- | --- | --- |
| *Target* | *Catalog Number* | | *Secondary detection* |
| Claudin-5 | Abcam #ab131259 | | Donkey α-rabbit:488 |
| Ferroportin | Novus #21502SS | | Donkey α-rabbit:647 |
| Nrf2 | Abclonal #A21176 | | Donkey α-rabbit:647 |
| Occludin | Abclonal #A2601 | | Donkey α-rabbit:488 |
| Transferrin Receptor | R&D Systems #AF2474 | | Donkey α-goat:488 |
| ZO-1 | Abclonal #A0659 | | Donkey α-rabbit:647 |
| **Secondary antibodies – Indirect Immunofluorescence** | | | |
| *Target* | | *Catalog Number* | |
| Donkey α-Rabbit:647 | | ThermoFisher #A-31573 | |
| Donkey α-Rabbit:488 | | ThermoFisher #A-21206 | |
| Donkey α-goat:488 | | ThermoFisher #A32814 | |
| Donkey α-mouse:488 | | ThermoFisher #R37114 | |

**
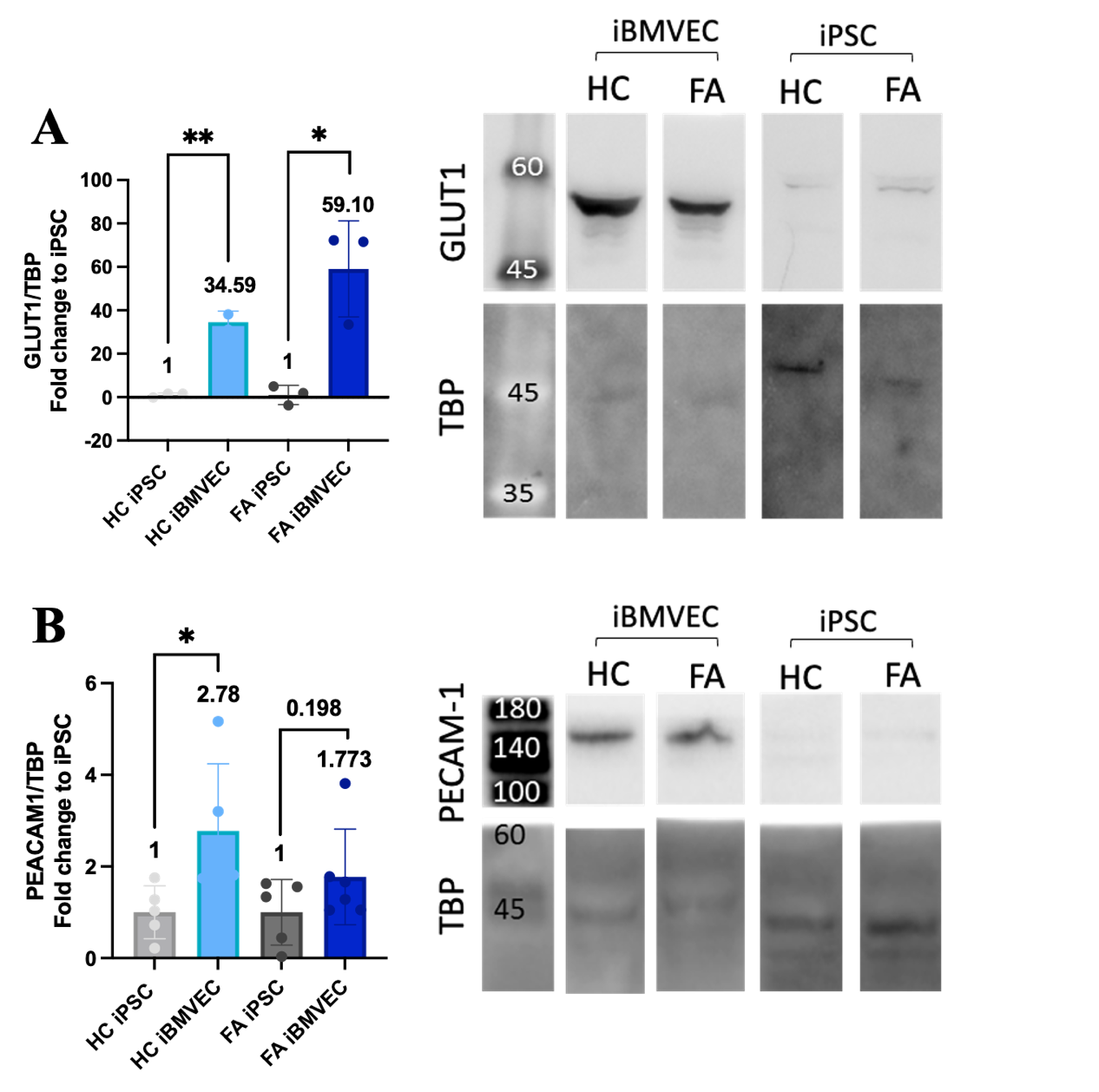
Supplemental Figures**

Supplemental Figure 1. Differentiation to iBMVEC induces upregulation of known EC markers GLUT-1 and PECAM-1. HC and FA iBMVEC and iPSC are electrophoresed and probed for (A) GLUT-1 and (B) PECAM-1 against the housekeeping gene TBP. Representative blots are shown to the right. Student’s T- test to each respective iPSC, α =0.05. *, *p*<0.05 and **, *p*<0.01. (A) n = 3 (both iPSC and FA iBMVEC) and n = 2 (HC iBMVEC). (B) n = 5 (both iBMVEC and HC iPSC) and n = 6 (FA iBMVEC).


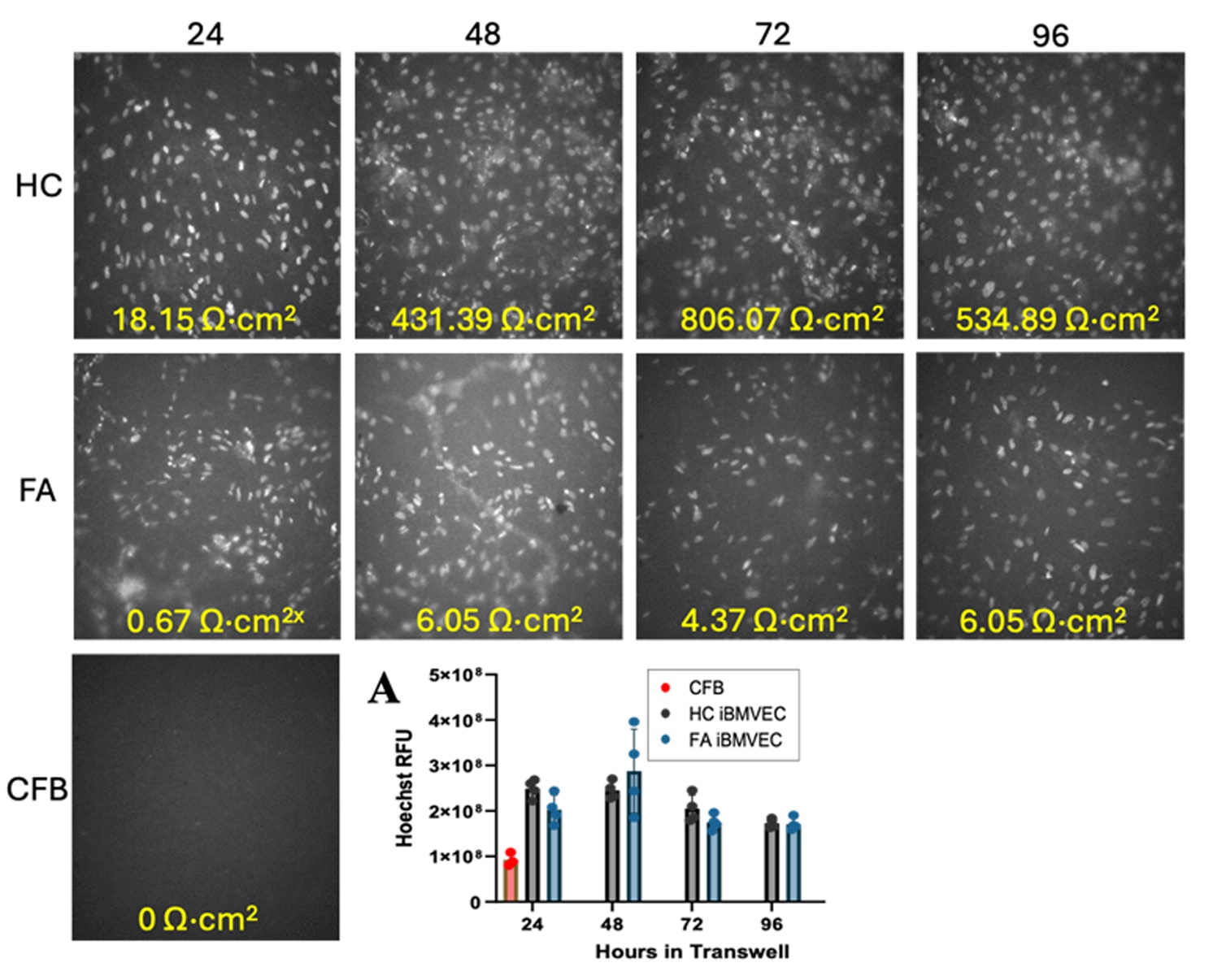


**Supplemental Figure 2. FA iBMVEC have equal coverage of the transwell compared to HC iBMVEC.** iBMVEC are seeded at 170,000 cells per transwell and media changed every 24 h. At each timepoint, transwells were analyzed for TEER (represented in yellow text), cells fixed in 3.7% paraformaldehyde and stained with 0.7ug/ml Hoechst. A cell-free blank (CFB) is used to assess background staining. (**A**) Hoechst staining is quantified per timepoint to assess potential changes in cell seeding density. Student’s T-test, α =0.05. All differences are non-significant. Biological replicates: (A) n = 3 (CFB) and n = 4 (HC and FA).
